# *GT198 (PSMC3IP)* germline variants in early-onset breast cancer patients from hereditary breast and ovarian cancer families

**DOI:** 10.18632/genesandcancer.132

**Published:** 2017-01

**Authors:** Stephanie Schubert, Tim Ripperger, Melanie Rood, Anthony Petkidis, Winfried Hofmann, Hildegard Frye-Boukhriss, Marcel Tauscher, Bernd Auber, Ursula Hille-Betz, Thomas Illig, Brigitte Schlegelberger, Doris Steinemann

**Affiliations:** ^1^ Department of Human Genetics, Hannover Medical School, Hannover, Germany; ^2^ Department of Obstetrics and Gynecology, Hannover Medical School, Hannover, Germany; ^3^ Hannover Unified Biobank, Hannover Medical School, Hannover, Germany

**Keywords:** early-onset breast cancer, *GT198*

## Abstract

*GT198*, located 470 kb downstream of *BRCA1*, encodes for the nuclear PSMC3-interacting protein, which functions as co-activator of steroid hormone-mediated gene expression, and is involved in RAD51 and DMC1-mediated homologous recombination during DNA repair of double-strand breaks. Recently, germline variants in *GT198* have been identified in hereditary breast and ovarian cancer (HBOC) patients, mainly in cases with early-onset. We screened a cohort of 166 *BRCA1/2* mutation-negative HBOC patients, of which 56 developed early-onset breast cancer before the age of 36 years, for *GT198* variants. We identified 7 novel or rare *GT198* variants in 8 out of 166 index patients: c.-115G>A (rs191843707); c.-70T>A (rs752276800); c.-37A>T (rs199620968); c.-24C>G (rs200359709); c.519G>A p.(Trp173*); c.537+51G>C (rs375509656); c.*24G>A. Three out of 7 identified variants (c.-115G>A, c.519G>A and c.*24G>A) with putative pathogenic impact were found in HBOC patients with breast cancer onset at ≤ 36 years. The nonsense mutation c.519G>A p.(Trp173*) was located within the DNA binding domain of GT198 and is predicted to induce nonsense-mediated mRNA decay. Functional analyses of c.-115G>A, and c.*24A>G indicated an influence of these variants on gene expression. This is the second study that gives evidence for an association between pathogenic *GT198* germline variants and early-onset breast cancer in HBOC.

## INTRODUCTION

Roughly 5-10% of all breast and ovarian cancers occur in the context of genetic predisposition [1]. Pathogenic mutations in *BRCA1* and *BRCA2* account for approximately 25% of all cases of hereditary breast and ovarian cancer (HBOC) [1]. HBOC is characterized by an autosomal dominant inheritance pattern with incomplete, age-dependent penetrance, variable expressivity, an early age of breast cancer onset, and/or a positive family history with first and second degree relatives affected with breast and/or ovarian cancer [2].

Since the identification of *BRCA1* [3] and *BRCA2* [4] in 1994 as the two main HBOC-susceptibility genes, further genes involved in DNA-repair mechanism and/or predisposing for other rare cancer predisposing syndromes have been identified as additional low or moderate risk factors for HBOC (e.g. *ATM, BARD1, BRIP1, CDH1, CHEK2, NBN1, PALB2. RAD51C*, *RAD51D, RECQL* and *TP53*) [1,5–15]. However, genetic testing of these HBOC risk genes, including *BRCA1* and *BRCA2*, detects causative variants in no more than 40% of the families with HBOC [1,16–17]. Thus, in the majority of cases, the HBOC predisposing genetic factors remain unknown.

*GT198* has been described as a novel potential candidate gene for early-onset breast and ovarian cancer by Peng *et al.* [18]. *GT198,* also known as *PSMC3IP*, *TBPIP* (Tat binding protein interacting protein), and *HOP2* (ortholog of *S. cerevisiae* Hop2), has been mapped 470 kb proximal of *BRCA1* on chromosome 17q21 [19,20]. It encodes for the *PSMC3* (proteasome 26S subunit, ATPase, 3)-interacting protein, which is strongly expressed in adult testis and, at much lower levels, in other tissues, such as ovary and mammary gland. It acts as a transcriptional coactivator by interacting with the DNA-binding domains of nuclear receptors, such as estrogen receptor alpha and beta, thyroid hormone receptor beta 1, androgen receptor, glucocorticoid receptor, and progesterone receptor [21]. Furthermore, GT198 has been shown to stimulate RAD51 or meiotic DMC1-mediated DNA strand exchange during repair of DNA double-strand breaks [22–24]. GT198 also has an anti-apoptotic role by repressing caspase 8 activity in estrogen receptor-positive and triple-negative breast cancer cells [25].

In 2011, *GT198* has been described as a novel candidate gene for primary ovarian insufficiency, when a homozygous 3 bp in-frame deletion in exon 8 (NM_016556.3, c.600_602del; p.Glu201del) was found in five affected females of a consanguineous Palestinian family with XX-female gonadal dysgenesis [26]. However, no association of mutated *GT198* with primary ovarian insufficiency has been found in a cohort of 50 patients with Swedish ethnicity [27]. Subsequently, potential pathogenic germline variants in *GT198* were identified at a low frequency in patients with HBOC, mostly with early cancer onset and in one patient with apparently sporadic early-onset breast cancer [18]. Deleterious somatic variants, which often cluster in the 5´-UTR and at the exon 4/intron 4 border of *GT198,* are abundantly detectable in breast and ovarian cancers and in fallopian tube tumors [18,24,28,29]. In order to evaluate the role of GT198 in HBOC, we screened 166 *BRCA1/2* mutation-negative patients, who fulfilled the diagnostic criteria of the German Consortium of Familial Breast and Ovarian Cancer (criteria details see Supplementary Table 1). Fifty-six of them developed breast cancer before the age of 36 years and, thus, were regarded as early-onset breast cancer patients (≤ 35 years). *GT198* variants were investigated regarding their functional impairment.

## RESULTS

**Figure 1:**
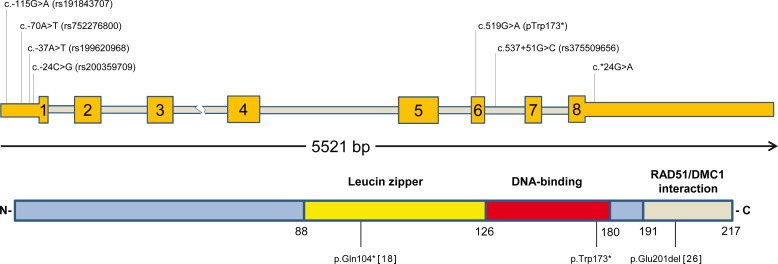
Schematic illustration of the identified mutations in human *GT198* Coding exonic regions of *GT198* (exons 1-8) are indicated by yellow boxes, while untranslated regions are highlighted as yellow bars and introns as grey bars. Detected *GT198* variants are indicated above, using the HGVS nomenclature guidelines (http://varnomen.hgvs.org/) and reference NM_016556.3. The classification of GT198 functional domains was made in accordance to references [18] and [23]. Previously identified pathogenic germ line variants are also shown [18,26].

**Figure 2:**
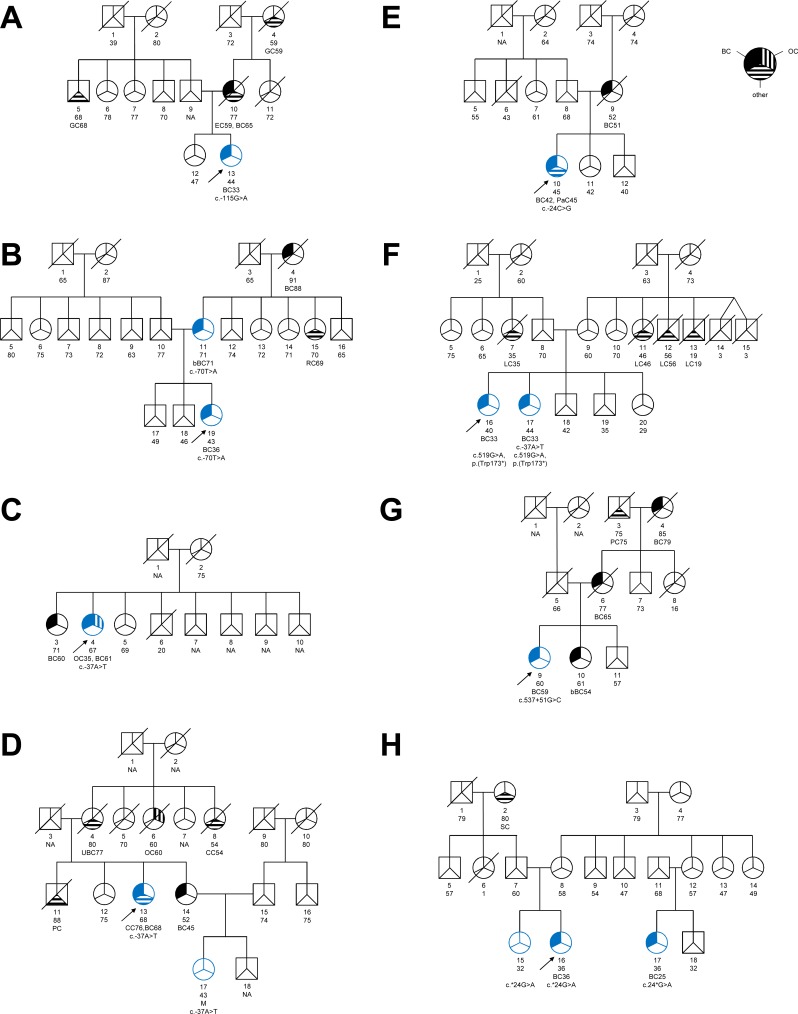
Pedigrees of families with *GT198* germline variants We identified 8 different families termed as **A-H** with *GT198* germline variants. A-H: Family pedigrees, circles, females; squares, males. Breast cancer (BC) affected individuals are marked by upper left corner filled symbols; cases with ovarian cancer (OC) are highlighted as cross-striped areas in the upper right corner; b, bilateral; other cancer types are shown in the lower third of the symbol as striped region and are abbreviated as follows: CC, colon cancer; EC, endometrial cancer; GC, gastric cancer; LC, lung cancer; M, meningioma; PaC, pancreas carcinoma; PC, prostate cancer; RC, renal carcinoma; SC, skin cancer; UBC, urinary bladder cancer. Unfilled symbol, unaffected relative; slashed symbol, indicate deceased family member; numbers below symbols are individual identifier, followed by information about the age at death, age of healthy individual and age of affected individual, while the age of cancer diagnosis is listed below. NA: unknown age. For *GT198* variant tested members are shown in blue symbols, and the respective *GT198* change is shown below. The index case is marked by an arrow.

A germ line nonsense mutation in *GT198* has been identified in a family with hereditary breast and ovarian cancer and early-onset breast cancer and in another unrelated case with early-onset breast cancer [18]. This report prompted us to screen 166 HBOC-affected index patients, 56 of them showing early-onset breast cancer, for *GT198* variants (Supplementary Table 1). We found rare or novel *GT198* variants with possible pathogenic significance in 8 unrelated index cases with a family history of breast and/or ovarian cancer. (Table 1, Figure 1, Figure 2). Seven patients with *GT198* variants were affected with breast cancer with a median age of cancer onset of 36 years, and one heterozygous index case was diagnosed with ovarian cancer at the age of 35 years. *GT198* variants were identified in 2 out of 56 early-onset breast cancer cases (3.6%) and in 6 out of 110 breast and ovarian cancer patients (5.5%) with suspected HBOC diagnosis without early-onset (Table 1).

**Table 1:** GT198 variants in breast and ovarian cancer cases

GT198 variant	rs-nmnber	localisation	family-ID	Index	cancer of index with age of onset (y)	neoplasia in other relatives	FFPE analyses
c.-115G>A	rsl 91843707	5′-UTR	A	A13	BC (DCIS, ER-,PR-, HER+)(33y)	A4: GC; A5: GC;A10: lob. BC, EC	
c.-70T>A	rs752276800	5′-UTR	B	B19	BC (DC, ER+,PR+, HER-) (36y)	B4: BC (LCIS); Bll: bBC (DC, DCIS, ER+.PR+.HER−); B15: RC	B19: BC; Bll: BC
c.-37A>T	rsl 99620968	5′-UTR	C	C4	OC (35y) BC (DC, ER+,PR+, HER-) (61y)	C3: BC	C4: BC
			D	D13	BC (68y), CC (76y)	D4: UBC; D6: OC; D8: CC; Dll: PC; D14: BC; D17:M	
c.-24C>G	rs200359709	5′-UTR	E	E10	BC (DC, ER+,PR+) (42y), PaC (45y)	E9: BC (DC)	E10: BC
c.519G>A, p.(Trpl73*)	none	exon 6	F	F16	BC (DC) (33y)	F7: LC; F11: LC; F12: LC; F13 -.LC-, FI 7: BC (DC, ER+.PR+, HER-); is also heterozygous for c.-37A>T	F16: BC; F17: BC
c. 537+51G>C	rs375509656	intron 6	G	G9	BC (ER+,PR-, HER-) (59y)	G3: PC; G4: BC; G6: BC; G10: bBC (DC, ER+,PR+,HER+)	G9: BC
c.*24G>A	none	3′-UTR	H	H16	BC (DC, ER+,PR+, HER-) (36y)	H2: SC -,H17:BC (DC, ER+.PR+, HER-)	

Breast cancer (BC); Ovarian cancer (OC); b, bilateral; DC, invasive ductal breast carcinoma; DCIS, ductal carcinoma *in situ*; LCIS, lobular carcinoma *in situ*; lob. BC, lobular breast cancer; CC, colon cancer; EC, endometrial cancer; GC, gastric cancer; LC, lung cancer; M, meningioma; PaC, pancreas cancer; PC, prostate cancer; RC, renal carcinoma; SC, skin cancer; UBC, urinary bladder cancer; HER-: HER2 overexpression-negative; HER+, HER2 overexpression-positive; PR+: progesterone receptor-positive BC; PR-: progesterone receptor-negative BC; ER+: estrogen receptor-positive BC; ER-: estrogen receptor-negative BC; HER, ER and PR status were obtained from archival medical reports. FFPE analyses: listed the cases in which mutation analyses of *GT198* with genomic DNA from archived formalin fixed paraffin embedded tumors were also performed. Family members, in which segregation analysis for the respective variants were performed are italicized.

We identified 1 common (rs2292752, c.338-15C>G) and 5 rare nucleotide substitutions (rs191843707 (c.-115G>A), rs752276800 (c.-70T>A), rs199620968 (c.-37A>T), rs200359709 (c.-24C>G) and rs375509656 (c.537+51G>C)). These 5 variants were listed in the European population in public databases with allele frequencies of <1% (Exome Aggregation Consortium and the NCBI data base, including the 1000 Genome Project). We observed a Hardy-Weinberg equilibrium for all detected variants in cases and controls, with the exception of the common variant rs2292752 (c.338-15C>G), which was in disequilibrium in controls. We found a significant difference for the allele frequencies of rs752276800 (c.-70T>A) and rs375509656 (c.537+51G>C) between cases and controls (Table 2).

**Table 2:** Genotype and allele frequency of *GT198* variants in *HBOC*-cases and controls

*GT198* variant	E1BOC cases (n=166) Genotype frequencies	HWE	Controls Genotype frequencies	HWE	E1BOC cases Allele frequencies	Controls Allele frequencies	ASSOC
c.-115G>A rsl 91843707	G/G=165 (99,4%) G/A=l (0,6%)	n.s.	G/G=12073 (98,72%) G/A=156 (1,28%)	n.s.	G=331 (99,7%) A=1 (0,3%)	G=24302 (99,36%) A=156 (0,64%)	n.s.
c.-70T>A rs752276800	T/T=165 (99,4%) T/A=l (0,6%)	n.s.	T/T=24352 (99,99%) T/A=l (0,01%)	n.s.	T=331 (99,7%) A=1 (0,3%)	T=48705 (99,99%) A=1 (0,01%)	0,01349
c.-37A>T rsl 99620968	A/A=164 (98,8%) A/T=2 (1,2%)	n.s.	A/A=31569 (99,58%) A/T=134 (0,42%)	n.s.	A=330 (99,4%) T=2 (0,6%)	A=63272 (99,79%) T=134 (0,21%)	n.s
c.-24C>G rs200359709	C/C=165 (99,4%) C/G=l (0,6%)	n.s.	C/C=1315 (99,92%) C/G=l (0,08%)	n.s.	C=331 (99,7%) G=1 (0,3%)	C=2631 (99,96%) G=1 (0,04%)	n.s.
c.338-15C>G rs22 92752	C/C=27( 16,27%) C/G=87 (52,4%) G/G=52 (31,33%)	n.s.	C/C=7085 (21,26%) C/G=16252 (48,77%) G/G=9985 (29,97%)	0,0018	C=141 (42,47%) G=191 (57,53%)	C=30422 (45,65%) G=36222 (54,35%)	n.s.
c. 537+51G>C rs375509656	G/G=165 (99,4%) G/C=l (0,6%)	n.s.	G/G=33367 (99,99%) G/C=l (0,01%)	n.s.	G=331 (99,7%) C=1 (0,3%)	G=66735 (99,99%) C=1 (0,01%)	0,009876

Test of Hardy-Weinberg deviation and allelic association of identified *GT198* variants between HBOC cases and controls. Genotype and allele frequencies of the Non-Finnish Europeans from the Exome aggregation consortium or the European population from the NCBI database (for rs200359709; Agilent ss#491737091) were used as controls. HWE: Hardy-Weinberg equilibrium, a p-value <0.05 after chi-square goodness of fit test with 1 degree of freedom indicates Hardy-Weinberg disequilibrium. ASSOC shows the p-value after Fisher’s exact test or Pearson´s Chi-squared test for rs2292752, n.s. non-significant p-value (p≥0,05).

We also detected a heterozygous nonsense mutation (c.519G>A; p.(Trp173*)) in exon 6 (Table 1), which was classified as disease causing by MutationTaster, presumably by inducing nonsense-mediated mRNA decay (NMD), and its localization within the DNA-binding domain of GT198 [18]. Since there are many GT198 isoforms the prediction was made also for the protein coding transcript variants ENST00000253789 (c.483G>A; p.(Trp161*), ENST00000587209 (c.330G>A; p.(Trp110*) and ENST00000590760 (c.144G>A; p.(Trp48*). The affected amino acid tryptophan is highly conserved among vertebrates (PhyloP:6,302, PhasCons:1). This truncating mutation is also listed in the COSMIC database (mutation ID 4431647) and has been detected as a somatic variant by exome sequencing in one patient with esophagus squamous cell carcinoma [30]. In our cohort, the nonsense mutation p.(Trp173*) was found in two sisters (F16 and F17), which were both diagnosed with unilateral breast cancer (invasive ductal carcinomas) at 33 years of age (Table 1, Figure 1, Figure 2). One of the sisters was also heterozygous for the c.-37A>T variant (rs199620968). The c.-37A>T substitution was also detected in two other unrelated cancer patients, each in the heterozygous state: in patient C4, which was diagnosed with ovarian cancer at the age of 35 years followed by unilateral invasive ductal breast cancer at the age of 61 years, and in index case D13, which was affected by unilateral breast cancer at the age of 68 (Table 1). The heterozygous substitution c.-37A>T was also present in the 43-year-old meningioma affected niece (D17) of index patient D13.

**Figure 3:**
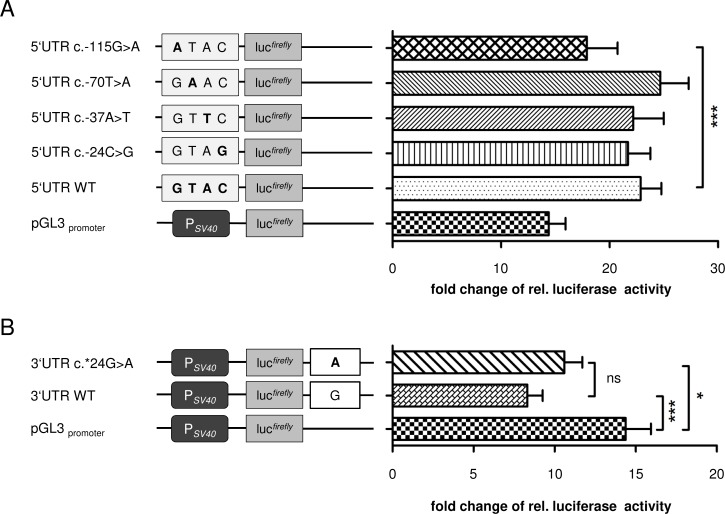
Luciferase assays of the *GT198* 5´- and 3´-UTR in transiently transfected HEK293T cells **A.** Relative luciferase activity of the 5′-UTR vectors. 5′UTRc.-115G>A, p.GL3.basic.5′UTR.c.-115G>A; 5′UTRc.-70T>A, p.GL3.basic.5′UTR.c.-70T>A; 5′UTRc.-37A>T, p.GL3.basic.5′UTR.c.-37A>T; 5′UTR.c.-24C>G, p.5′UTRc.-24C>G; 5′UTRWT, p.GL3.basic5′UTRWT. **B.** Luciferase assays of *GT198* p.3′UTRc*24G>A and the respective wildtype construct. 3′UTRc.*24G>A, p.GL3.promoter.3′UTR.c.*24G>A; 3′UTRWT, p.GL3.promoter.3′UTRWT. In (A) and (B), bar graphs show the mean+s.d. (*n* = 9 (three independent experiments performed in triplicates); pGL3.basic was set to 1; following D’Agostino and Pearson omnibus normality test, one-way ANOVA with post-hoc Tukey’s multiple comparison test was performed, *P* < 0.05).

As copy number gains of a mutated *GT198* allele with a nonsense mutation have recently been reported in a breast cancer affected patient [18], we additionally screened the index case F16 and her sister F17 for copy number changes by a custom-made 60k eArray. No copy number gains or losses of *GT198* and no further structural rearrangements were detected by array-CGH. From all identified substitutions in the 5′-UTR (i.e. rs191843707 (c.-115G>A), rs752276800 (c.-70T>A), rs199620968 (c.-37A>T) and rs200359709 (c.-24C>G)), the variant c.-115G>A was found once in our own study in a female that developed unilateral breast cancer at the age of 33 years (Table 1, Figure 1, Figure 2) and has previously been described in hereditary breast and ovarian cancer [18]. It was predicted that c.-115G>A deleteriously alters the binding site for the ETS domain-containing factor ELK1 at positions c.-111_-120 (Supplementary Figure 1). To evaluate whether the predicted effects on transcription factor-binding might influence *GT198* expression, luciferase assays were performed for all identified 5´-UTR variants in HEK293T cells (Figure 3). Transfection of the c.-115A construct showed a significant decrease of relative luciferase activity by 22% compared to the wild type allele (Figure 3). In contrast, for c.-70T>A, c.-37A>T, and c.-24C>G, no significant differences of luciferase activities were detected. *In silico* analysis of the intron 6 variant c.537+51G>C (Table 1) provided no evidence for altered splicing.

We further detected a novel nucleotide substitution within the 3′-UTR (c.*24G>A) of *GT198* in patient H16 diagnosed with unilateral breast cancer at the age of 36 years (Table 1, Figure 1, Figure 2). The variant is also present in her 32-year-old unaffected sister and in her cousin H17 diseased from breast cancer at the age of 25 years (Table 1, Figure 2). *In silico* analyses indicated that only less conserved microRNA-binding sites (hsa-miR-1224-3p, hsa-miR-1280, hsa-miR-2114, hsa-miR-2355-5p, and hsa-miR-4286) were affected by c.*24G>A. To investigate whether this position is important for microRNA-binding, luciferase assays according to Buurman *et al.* [31] were performed (Figure 3). A significant decrease of luciferase activity of approximately 42% was observed when the wildtype 3′-UTR was introduced downstream to the reporter gene (Figure 3). Luciferase activity in HEK293T cells transfected with the c.*24A construct was decreased by only 26% compared to the control, suggesting a negative effect of c.*24G>A on microRNA-binding and, thus, to an increased gene expression.

We further confirmed the variants c.-70T>A, c.-37A>T, c.-24C>G, c.537+51 G>C and the nonsense mutation p.(Trp173*) in FFPE-breast cancer samples of the respective index cases. For the variants c.-115G>A, and c.*24G>A, no tumor material was available (Table 1). As somatic variants are frequently observed in breast and ovarian cancers [28,29], we sequenced *GT198* in the available tumor samples (Table 1, Supplementary Table 2). No additional second hits were detected.

We further screened all 8 index cases carrying *GT198* variants for pathogenic variant in additional low or moderate risk genes for HBOC (e.g *ATM, CDH1, CHEK2, NBN1, PALB2*, *RAD51C*, *RAD51D,* and *TP53)* using the TruSight Cancer panel (Illumina, San Diego CA). No pathogenic variants were detected in any of these additional risk genes. In order to screen for HBOC predisposing copy number changes in the 8 carriers of *GT198* variants, a customized high-resolution 8×60k array (Design:069100, HBOC-2, Agilent technologies) for comparative genomic hybridization CGH covering the 94 genes of the TruSight cancer panel was used [32]. We identified no aberrant copy number changes in the HBOC cancer risk genes [5,8–13,15], including *BRCA1* and *BRCA2*, by high resolution array CGH.

## DISCUSSION

*GT198′s* location in a genomic region on 17q21, previously linked to hereditary breast and ovarian cancer, makes an association of *GT198* disease-causing changes with HBOC and sporadic early-onset breast cancer likely [18–20,24,33]. Germline variants with possible pathogenic impact have been found in HBOC cases with mostly early onsets (median age 35 years) and in an apparently sporadic case of breast cancer with an onset age of 30 years [18].

Eight out of 166 unrelated index cases (4.8%) in our study were heterozygous for rare or novel *GT198* variants with yet unknown impact on GT198 function, which is similar to the detection frequency of the first report, in which 8 out of 212 index patients (3.8%) have been heterozygous for putative pathogenic *GT198* germline variants [18]. Three out of 8 heterozygous carriers of *GT198* variants in the present study showed early-onset of breast or ovarian cancer (≤35 years), which is, albeit in lower frequency, congruent with the former findings of Peng *et al*. [18], who reported that 6 out of 8 index cases carrying *GT198* variants were affected by early-onset breast or ovarian cancer.

We identified two germ line variants in *GT198* (c.519G>A p.(Trp173*) and c.*24G>A), that were neither listed in the NCBI database nor in SNP data bases of the ExAc Browser and of EVS. The nonsense mutation c.519G>A; p.(Trp173*) is reported once in the COSMIC database and was detected in a human esophagus squamous cell carcinoma [30]. Our own *in silico* analysis suggest a negative impact of c.519G>A on GT198 expression and function. The induced premature translation termination codon of c.519G>A is located in the DNA binding domain of GT198, and is predicted to induce nonsense-mediated mRNA decay of the aberrant transcript [34]. This stop codon is located downstream of an alternative translation initiation codon within exon 5, that leads to the expression of a truncated protein isoform, harboring the DNA binding domain and the C-terminus [24]. The DNA binding domain is able to bind both single- and double-stranded DNA and is important for GT198 DNA repair activity after DNA double-strand breaks [18]. It has been shown by *in vitro* assays that especially the amino acid residues 171-178 of murine Hop2 at the C-terminus, which are 100% identical to the human orthologue, have a high affinity for single-stranded DNA [22], but it still remains unknown, whether amino acid residue 173 is indispensable for RAD51 single-stranded DNA presynaptic filament stabilization or homologous DNA pairing, which are both important for RAD51-mediated homologous recombination of damaged chromosomes [23]. Its presence in two breast cancer affected sisters that were both diagnosed having cancer at 33 years of age (Table 1, Figure 2), makes a positive association of c.519G>A with early-onset breast cancer likely. A pathogenic nonsense mutation (c.310C>T; p.(Q104*)) has already been identified in a former study by Peng *et al.* [18] in two unrelated female breast cancer patients that were diagnosed with breast cancer at the age of 30 and 33 years. The induced premature stop codon affects the leucine zipper dimerization domain of GT198, which is also required for protein-protein interaction and transcriptional regulation and has been shown in *in vitro* cell culture experiments to abolish RAD51-mediated DNA repair activity after γ-irradiation [18]. It is assumed that mutated and alternate transcripts are counteracting in a dominant negative manner with wildtype GT198 [18,24].

Some microRNA-binding sites are predicted in the 3′-UTR of *GT198*. The substitution c.24*G>A is located within a weakly-conserved microRNA-binding site for hsa-miR-1224-3p, hsa-miR-1280, hsa-miR-2114, hsa-miR-2355-5p, and hsa-miR-4286. However, it is currently unknown whether GT198 expression is regulated by one of these *in vivo*. Our own *in vitro* data points to an impaired effect of the variant c.24*G>A on microRNA-binding. Whether this would also affect GT198 expression *in vivo* requires further elucidation. The c.24*G>A substitution was found to segregate with early-onset breast cancer in an affected female maternal cousin, but was also present in a 32-year-old healthy sister of the index case (Table 1, Figure 2).

Interestingly, rare variants within the *GT198* 5′-UTR and 3′-UTR were frequently found in breast, ovarian and fallopian tube cancers [18,24,28,29]. Six out of 166 cancer patients of our cohort were heterozygous for rare or novel nucleotide substitutions located in the 5´-UTR or 3′-UTR of *GT198*. Two of these variants, c.-115G>A and c.-37A>T, have already been identified as hereditary variants in familial cases of breast and ovarian cancer [18]. Interestingly, one variant in the 5′-UTR (c.-37A>T) has been found in the germline of two unrelated familial cases in our own and in a former study [18]. One of the carriers of c.-37A>T was affected by ovarian cancer at early-onset (35 years). This variant has previously been presented as a somatic variant in serous ovarian carcinoma, fallopian tube cancer, and endometrial carcinoma [24]. All identified *GT198* 5′-UTR variants are also detectable in the general population, albeit at low allele frequency, and a possible disease association still remains unknown. Especially the substitution c.-115G>A is listed in the European population of the Exome Aggregation Consortium with an allele frequency of 0.64%. Our own *in silico* and *in vitro* data suggest no influence of c.-70T>A, c.-37A>T and c.-24C>G on GT198 expression. In contrast, the variant c.-115G>A induced a slightly, albeit significant, decrease of reporter gene expression in transiently transfected HEK293T cells. Our own *in silico* predictions led us to speculate if this effect could be mediated by destroying a binding site for the transcription factor ELK1 (ETS domain-containing protein Elk-1), a member of the ETS family of transcription factors, which regulates the expression of genes involved in cell proliferation, chromatin modelling and apoptosis [35,36]. ELK1 overexpression is frequently observed in many carcinomas, including breast cancer [36]. Recently, the MZF1/Elk-1 complex has been identified as mediator of protein kinase C alpha (PKCα) expression in triple-negative breast cancer, which induces cell migration and invasion of triple negative breast cancer cells and poor outcome [37]. However, the pathogenic effect of altered GT198 expression in general and the influence of c.-115G>A on GT198 expression in breast or ovary *in vivo* is still unknown and requires further elucidation.

The minor alleles c.-70T>A and c.537+51G>C were observed significantly more frequent in cases than in European controls of the ExAC database (Table 2). Our own analyses suggest a benign effect of both variants on GT198 function, and we, therefore, ascribe the discrepancies in allele frequencies to the small sample size of the analyzed case cohort.

We here present a screen for pathogenic changes in *GT198* in patients with *BRCA1/2*-negative HBOC. We identified seven different rare or novel *GT198* variants with yet unknown impact on GT198 function, of which six were absent or extremely rare in the ExAC database in Europeans. Three variants (c.-115G>A, c.519G>A and c*24G>A) found in familial breast cancer patients with early-onset at ≤ 36 years seem to have an impact on GT198 function and may contribute to breast cancer predisposition. GT198 participation in steroid hormone receptor-mediated gene expression, its function in DNA recombination, and its ability to stimulate RAD51 mediated DNA strand exchange [18,23,29], makes its implication in oncogenesis conceivable. Further, comprehensive mutation screenings in multi-national case and control collectives are required to evaluate the role of GT198 in breast and ovarian cancer predisposition.

## MATERIAL AND METHODS

### Study cohort

The analyzed cohort was composed of 166 unrelated, female breast and/or ovarian cancer patients of mixed Caucasian, mostly German origin, which were referred to our outpatient clinic between 2004 and 2014. All selected individuals, including familial cases (n=158) and early-onset breast cancer cases without a positive family history (n=8), fulfilled the inclusion criteria for *BRCA1* and *BRCA2* testing of the German Consortium for HBOC (Supplementary Table 1) [38]. All women have given their informed consent for participating in the study, which was approved by the hospital´s ethics committee (Hannover Medical School, ethic votum 4121). Comprehensive data about the family history, including data about breast and ovarian cancer development over at least three generations, tumor pathology, and about *BRCA1/2* mutational status were available for each case. All samples were previously shown to be negative for deleterious variants within *BRCA1* and *BRCA2* using routine diagnostic methods, including sequencing and multiplex ligation probe-dependent amplification (MRC Holland, Amsterdam, the Netherlands) for *BRCA1*. For 162 out of 166 samples structural aberrations affecting *BRCA2* were also excluded by MLPA and/or array-CGH analyses (Agilent Technologies, Santa Clara, United States). However, 5 samples were heterozygous for missense variants of unknown significance in *BRCA2* (ENST00000380152, c.831T>G, p.(Asn277Lys); c.995T>A, p.(Ile332Asn); c.4782G>T, p.(Met1594Ile); c.6101G>A, p.(Arg2034His); c.7562T>C p.(Ile2521Thr)).

The cohort encompasses 155 individuals with breast cancer (135 unilaterally and 20 bilaterally affected women), 9 individuals with ovarian cancer and 2 individuals with breast and ovarian cancer. Age at diagnosis of breast cancer ranged from 17 to 68 years (median 39 years), while the age of onset for ovarian cancer vary from 21 to 67 years (median 33.5 years). In 56 out of 155 breast cancer patients, diagnosis was made before 36 years. The majority of index patients (n=90) originated from families with at least two first and/or second degree female relatives affected by breast cancer, of whom one individual was diagnosed before age of 51 years. For 32 patients, there was a family history of at least one female breast and one ovarian cancer or one woman with breast and ovarian cancer. Thirty one samples were derived from families with at least one woman diagnosed with bilateral breast cancer before age of 51 years. Eight patients developed early-onset (≤ 35 years) breast cancer and had no familial history for HBOC. Three patients had a family history with at least 3 first or second degree relatives with breast cancer and two recruited index patients had a family history with at least two ovarian cancers (Supplementary Table 1).

### DNA extraction and sequencing

Genomic DNA from EDTA blood samples and buccal mucosa smears were extracted using QIAamp DNA Blood Midi and Mini Kit (Qiagen, Hilden, Germany), respectively. From selected breast cancer cases, genomic DNA from formalin-fixed and paraffin-embedded (FFPE) tumor tissues was extracted from 5 μm serial sections using GeneRead DNA FFPE Kit (Qiagen) according to the manufacturer’s instructions. All tissue samples were previously histologically examined by pathologists. A hematoxylin and eosin-stained section of each tumor paraffin block was histologically examined to define the area with 15-80 % tumour cells to be macro-dissected for DNA extraction.

All 8 *GT198* exons encompassing the entire coding exons, adjacent intronic regions and parts of the flanking 5´-UTR and 3´-UTR were PCR amplified and subsequently sequenced using an ABI genetic analyzer 3130xl (Applied Biosystems, Darmstadt, Germany). For FFPE samples a set of additional primers was used. Primers were designed using the software Primer3 (http://bioinfo.ut.ee/primer3-0.4.0/primer3/) (Supplementary Table 2). Illustra ExoProStar 1-Step (GE Healthcare, Munich, Germany) was used for PCR product purification before sequencing PCRs. Finally, sequencing products were cleaned up by Sephadex G-50 purification (Sigma-Aldrich Chemie GmbH, Steinheim, Germany). Variant analyses were performed using Sequence Pilot version 4.3.0 (JSI Medical Systems, Ettenheim, Germany) and the NCBI sequence NM_016556.3 as reference.

The TruSight Cancer panel (Illumina, San Diego CA) was used for target enrichment of *ATM, CHEK2, CDH1, NBN1, PALB2, RAD51C*, *RAD51D* and *TP53* of selected index cases. Sequence analyses for the additional HBOC risk genes were performed with the module NextSeq of Sequence Pilot version 4.3.0 (JSI Medical Systems, Ettenheim, Germany) and the NCBI transcripts NM_000051 (*ATM)*, NM_007194.3 (*CHEK2)*, NM_002485 (*NBN1*), and the Ensembl transcripts ENST00000261769 (*CDH1*), ENST00000261584 (*PALB2*), ENST00000337432 (*RAD51C*), ENST00000345365 (*RAD51D*) and ENST00000269305 (*TP53*) as references.

### Variant and statistical analyses

All identified rare variants were validated by PCR amplification and sequencing of an independent DNA sample.

Detailed *in silico* predictions for identified *GT198* variants were made with tools implemented in *alamut visual* (Align GVGD, SIFT, MutationTaster, Polyphen2) (interactive biosoftware, *Version 2.7 rev.1,* Rouen, France) [39–42]. Data from the European population retrieved from the ExAC Browser (http://exac.broadinstitute.org/) or the NCBI database (https://www.ncbi.nlm.nih.gov/nuccore) and the European-African population from the exome variant server (http://evs.gs.washington.edu/EVS/) served as controls. Variants within the 5´-UTR were analyzed for putative effects on transcription factor binding using “JASPAR” (version 5.0_ALPHA, http://jaspar.binf.ku.dk/) and PROMO (http://alggen.lsi.upc.es/cgi-bin/promo_v3/promo/promoinit.cgi?dirDB=TF_8.3). For splice analyses the programs Berkeley Drosophila Genome Project (http://www.fruitfly.org/seq_tools/splice.html), and ASSP (http://wangcomputing.com/assp/index.html) were used. Additionally, a splice analysis using *alamut visual (interactive biosoftware)* with the included tools SpliceSiteFinder-like, MaxEntScan, NNSPLICE, GeneSplicer and Human Splicing Finder (HSF2.4.1) was also performed. MicroRNA-binding site predictions were made with the program Alamut visual based on miRanda predictions and microRNA.org targets. The effect of the nonsense mutation was evaluated by the program MutationTaster (www.mutationtaster.org) [42].

To evaluate whether the genotype distribution of *GT198* variants of cases and controls were in Hardy-Weinberg equilibrium (HWE), a chi-squared goodness-of-fit test with one degree of freedom was performed. For HWE analysis the online software tool http://www.koonec.com/k-blog/2010/06/20/ and hardy-weinberg-equilibrium-calculator/from Strom and Wienker were used (http://ihg2.helmholtz-muenchen.de/cgi-bin/hw/hwa1.pl). Allele frequencies between cases and controls were compared with Fisher´s exact test for low number of individuals carrying the rare allele (≤5) or with Pearson’s chi squared goodness-of-fit test (R-software, https://www.r-project.org/). P-values of these comparisons were assessed descriptively, and defined to be statistically significant if p<0.05. As control, datasets (genotype and allele frequencies) for the European non-Finnish population retrieved from the ExAC Browser (http://exac.broadinstitute.org/) were used.

### Cloning and Luciferase assays

To investigate the effects of c.-115G>A, c.-70T>A, c.-37A>T, c.-24C>G and c.*24G>A on gene expression, luciferase reporter assays were performed in HEK293T cells. To subclone genomic regions immediately 5′ of the start and 3′ of the stop codon of *GT198*, specific primers tagged with sequences containing restriction enzyme sites were used for amplification (Supplementary Table 2). Primers GT198_5′UTR_XhoI_f and GT198_5′UTR_ HindIII_r were used to amplify a 564 bp genomic fragment on the corresponding patients’ DNA (A13, B19, D13, E10, Table1, Figure 2). Following an *Xho*I/*Hind*III double digestion, the fragment was integrated upstream of the firefly luciferase gene of the pGL3.basic vector (Promega, Mannheim, Germany). Primers GT198_3′UTR_XbaI_f and GT198_3′UTR_SpeI_r were used for amplification of a 1,070 bp amplicon on the corresponding patients’ DNA (H16, Table1, Figure 2). Amplicon and pGL3.promoter vector (Promega, Mannheim, Germany) were cut with *Xba*I and the amplicon was subcloned downstream of the firefly luciferase gene. Inserts of the obtained vectors containing mutant or wildtype alleles were evaluated by sequencing using an ABI genetic analyzer 3130xl (Applied Biosystems). Thus, seven different vectors containing either the wildtype (p.GL3.basic.5′UTR.WT and p.GL3. promoter.3′UTR.WT) or the rare *GT198* alleles (p.GL3. basic.5′UTR.c.-115G>A, p.GL3.basic.5′UTR.c.-70T>A, p.GL3.basic.5′UTR.c.-37A>T, p.GL3.basic.5′UTR.c.-24C>G, and p.GL3.promoter.3′UTR.c.*24G>A) were generated.

HEK293T cells were cultured in Dulbecco´s modified Eagle´s medium supplemented with 1 mM sodium pyruvate, 10% heat-inactivated fetal bovine serum, 100 units/ml penicillin and 100 μg/ml streptomycin in a humidified atmosphere with 5% CO at 37°C. For each of the seven firefly luciferase reporter constructs triplicates of 8,000 HEK293T cells were seeded in a 96-well plate in 100 μl medium. After 24 hours, cells were cotransfected with 25 ng luciferase reporter plasmid and 2.5 ng pGL-4.70 (Promega) using Lipofectamine 2000 (Invitrogen, Paisley, UK). Twenty-four hours after transfection, cells were lysed and firefly and renilla luciferase activity were measured using the Dual-Glo^®^ Luciferase Assay System (Promega) and a Synergy 2 Multi-Mode Microplate Reader (BioTek, Winooski, VT) in accordance to the manufacturer´s instructions.

### eArray-CGH analysis

EDTA blood-derived genomic DNA (average DLRS value 0,1) of F16 and F17, carrying the *GT198* truncating mutation p.(Trp173*) (Table 1), was further screened for copy number changes and structural rearrangements affecting *GT198* using a custom-made 60k eArray (Design:081270, brca1-2region, Agilent Technologies) with high resolution in the genomic regions on chromosome 17 (chr17:40,600,000-41,756,000, 1.2 Mb, GRCh37/hg19) and chromosome 13 (chr13:32,414,344-33,490,000, 1.1 Mb, GRCh37/hg19), with an average probe spacing of 300 bp. The *GT198* gene was covered by 85 probes.

Selected index cases (n=8) carrying putative pathogenic *GT198* variants were also screened for HBOC predisposing copy number variations by a customized high resolution 60k eArray (Design:069100, HBOC-2, Agilent technologies) [32]. Array CGH analysis was performed as recommended by the manufacturer. The female human DNA EA-100F was used as control (Kreatech Biotechnology, Amsterdam, The Netherlands). Fluorescence signals were scanned using a Dual Laser Scanner G2565CA (Agilent Technologies). Raw data analysis was performed using Feature extraction version 11.0.1.1 (Agilent Technologies). For further data analysis, Genomic Workbench 7.0.4.0 (Agilent Technologies) was used: ADM-2 algorithm, threshold 6, and no aberration filter for the brca1-2region, while a 2log0.2 filter was used for the HBOC-2 design.

## SUPPLEMENTARY MATERIALS FIGURE AND TABLES


