# Supplementary material for: GT198 (PSMC3IP) germline variants in early-onset breast cancer patients from hereditary breast and ovarian cancer families
Source: Genes Cancer. 2017 Jan;8(1-2):472–83. doi: 10.18632/genesandcancer.132 (PMC5369655; doi:10.18632/genesandcancer.132)
Supplement: Supplementary file 1 [file ganc-08-472-s001.docx]

**GT198 (PSMC3IP)** germline variants in early-onset breast cancer patients from hereditary breast and ovarian cancer families – Schubert et al.


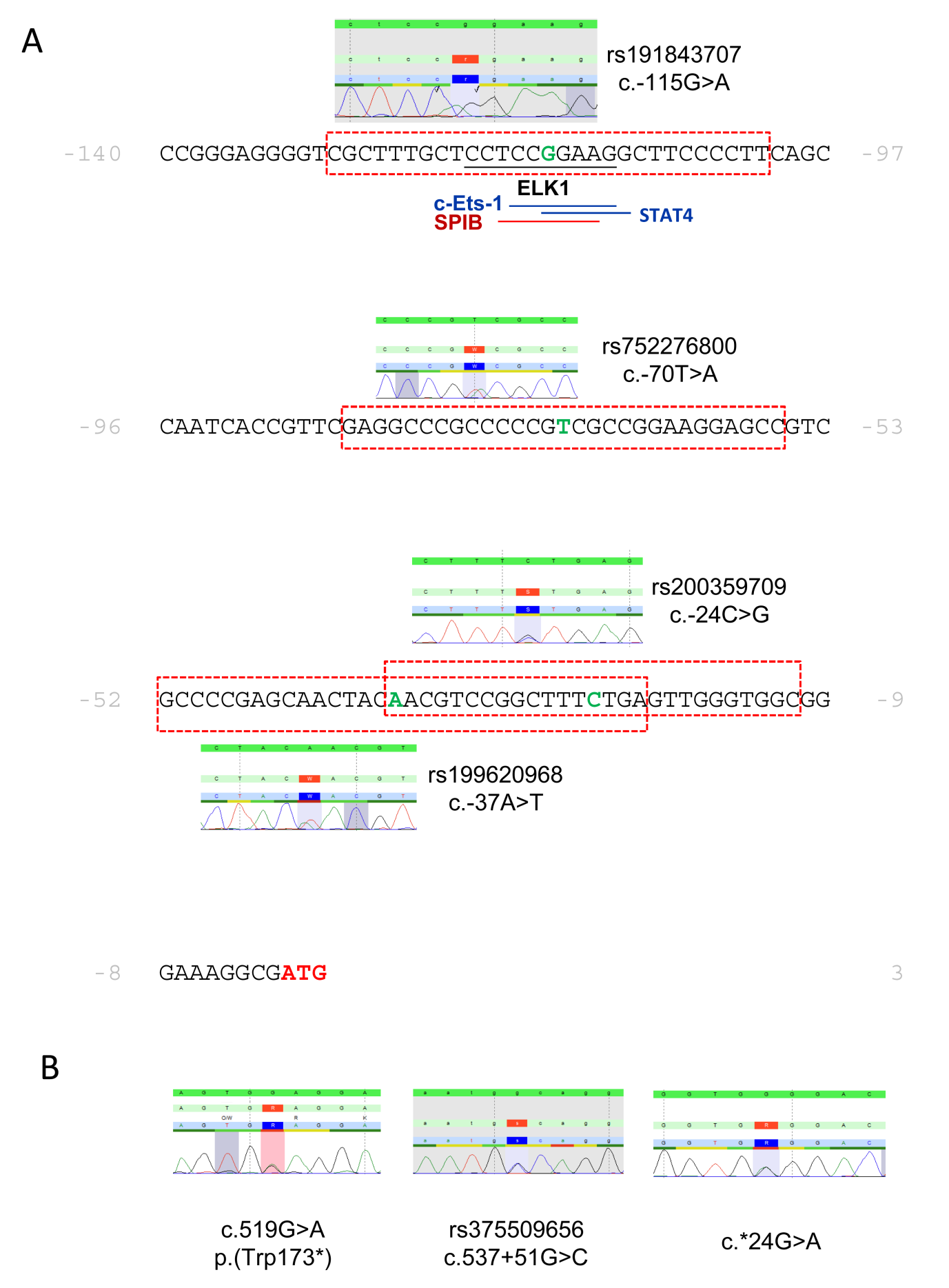


Supplementary Figure 1: Identified germ line variants in *GT198*.

1. The 5’-UTR sequence c.-140_-1 of *GT198* (NM_016556.3) is given in black letters in front of the start codon given in red. Nucleotides substituted in rs191843707, rs752276800, rs199620968 and rs200359709 are highlighted in green. The identified variants and the respective electropherograms are shown above and for rs199620968 below the sequence. Genomic sequence used for prediction of transcription factor binding sites within the prediction tools PROMO (cut off E value ≤0,002) and JASPAR (relative binding score of ≥0,9) are shown as red dashed boxes. Predictions for the reference sequence and the respective minor allele were made for each of the four variants. Effects on transcription factor binding sites (only losses or gains) that were predicted in both programs, in PROMO, and in JASPAR are labeled by black, blue, and red lines, respectively. The loss of an ELK1 binding site at c.-111_-120 was predicted for the minor allele of rs191843707 by JASPAR and PROMO. In addition, the single nucleotide substitution c.-115G>A was also predicted to destroy binding sites for c-Ets-1 (c.-111_-117) and STAT4 (c.-115_-110) by PROMO and for SPIB (c.-112_-118) by JASPAR.
2. Electropherograms for the identified nonsense mutation c.519G>A in exon 6, rs375509656 in intron 6, and c.*24G>A in the 3’-UTR.

**Supplementary Table 1: Familial history distribution of breast and ovarian cancer patients**

| Familial constellation | number of cases |
| --- | --- |
| ≥2BC, 1≤50 | 90 |
| ≥1BC and ≥1OC or 1BCOC | 32 |
| ≥1bBC≤50 | 31 |
| 1BC≤35 | 8 |
| ≥3 BC≥ 51 | 3 |
| ≥2OC | 2 |

Index patients recruited to the study were categorized in six groups with respect to their family history of breast and ovarian cancer. Groups are defined as follows: ≥2BC, 1≤50, ≥ 2 first and/or second degree breast cancer-affected female relatives, one of which was diagnosed before age 51 (n = 90); ≥1BC and ≥1OC or 1BCOC, ≥ 1 female breast and ≥ 1 ovarian cancer or ≥ 1 female with breast and ovarian cancer (n = 32); ≥1bBC≤50, bilateral female breast cancer, initial diagnosis before age 51 (n = 31); 1BC≤35, early-onset (≤ 35 years) female breast cancer, no family history of breast and ovarian cancer (n = 8); ≥3 BC≥ 51, ≥ 3 first and/or second degree affected relatives with female breast cancer (n=3); ≥2OC, ≥ two ovarian cancer affected relatives (n=2).BC: breast cancer, OC: ovarian cancer.

**Supplementary Table 2: Primers used for amplification and sequence analyses of human *GT198.***

| **Name** | **Sequence (5'→ 3')** | **Direction** |
| --- | --- | --- |
| GT198_5‘UTR_XhoI_f* | 5’-CTTGCTCGAGGGTGACATGCCACTGCGC-3‘ | forward |
| GT198 1F*^P^ | 5’-CCGGCCCGAATGCGCAAGTT-3‘ | forward |
| GT198-1.2F^P^ | 5’-CTTCAGCCAATCACCGTTCG-3‘ | forward |
| GT198-1.1R^P^ | 5’-GTAGTTGCTCGGGGCGACG-3‘ | reverse |
| GT198_5‘UTR_HindIII_r* | 5’-CGCGAAGCTTCGCCTTTCCCGCCACCC-3 | reverse |
| GT198-2.1F* | 5’-CGTGGGCGCGGGGAAGA-3‘ | forward |
| GT198-1.2R^P^ | 5’-GTCTTCCCCGCGCCCACG-3‘ | reverse |
| GT198-1.3R^P^ | 5’-CTGCAGGTACCTCAGGAGGA-3‘ | reverse |
| GT198-2F* | 5’-AGGTACCTGCAGGAGCAGAAC-3‘ | forward |
| GT198-2.1R^P^ | 5’-AACACATCCTGGGAGCTGTA-3‘ | reverse |
| GT198-2.2R^P^ | 5’-AGTCCGACGGAGAGGGAATC-3‘ | reverse |
| GT198-3.1F^P^ | 5’-CACATCACTGGCTGGATTTC-3‘ | forward |
| GT198-3.2F^P^ | 5’-CAACAAGGCAAGATCAAAGAG-3‘ | forward |
| GT198-1-2R* | 5‘-GAAATCCAGCCAGTGATGTG-3‘ | reverse |
| GT198-3.1R^P^ | 5’-CCGCAAAATAGATCTTCTGC-3‘ | reverse |
| GT198-3.2R^P^ | 5’-AAAGAGTTCACACCAGGAGT-3‘ | reverse |
| GT198-2-3R* | 5‘-GTAAATCTGGAAACCTCGTGAAGT-3‘ | reverse |
| GT198-4Fn*^P^ | 5‘-CCTGAGCATATCCAAATGGTGCC-3‘ | forward |
| GT198-4.1F^P^ | 5‘-GATGCTGACCTTCAAGTCCT-3‘ | forward |
| GT198-4.1R^P^ | 5‘-GCTCTGCACCTTAGCAGTGAGG-3‘ | reverse |
| GT198-4.2R^P^ | 5‘-CCTCTAGAATGAACCTAAGCC-3‘ | reverse |
| GT198-4Rn* | 5‘-GCAAAGGGGTCTTAGCTGCAAC-3‘ | reverse |
| GT198-5Fn*^P^ | 5‘-GATCCAGAGCAAGGTGGAACAC-3‘ | forward |
| GT198-5.1F^P^ | 5‘-GGTAGCAAGTGACCCCAG-3 | forward |
| GT198-5.2F^P^ | 5‘-CTGGCTACAGAGAGAGATTG-3 | forward |
| GT198-5.1R^P^ | 5‘-GAGTCACATGATTGGTAGCT-3’ | reverse |
| GT198-5.2R^P^ | 5‘-TGATGTTCACTCTGGACCTG-3‘ | reverse |
| GT198-6-7F*^P^ | 5‘-CAGGTCCAGAGTGAACATCA-3‘ | forward |
| GT198-6.1R^P^ | 5‘-AGCTCCCACACACTTACCAT-3’ | reverse |
| GT198-5Rn* | 5‘-TGGGCACTGCCTGTCAGTTT-3’ | reverse |
| GT198-6.2R^P^ | 5‘-TGGGCACTGCCTGTCAGTTT-3’ | reverse |
| GT198-7.1F^P^ | 5‘-AAACTGACAGGCAGTGCCCA-3’ | forward |
| GT198-8Fn*^P^ | 5‘-TCTTGTCAGGCTACAGAGCT-3‘ | forward |
| GT198_3‘UTR_XbaI_f* | 5‘-GAACTCTAGAGGGGCCCACGGTCAGGAC-3‘ | forward |
| GT198-8.2R^P^ | 5‘-CAAAAACAAGGTAGCCAGTGC-3‘ | reverse |
| GT198-6-7Rn*^P^ | 5‘-TCATCCGTCTCTATCCCAAC-3‘ | reverse |
| GT198-8.3F^P^ | 5‘-GCAGGATGTCAGAAGAGTGA-3‘ | forward |
| GT198-8R^P^ | 5‘-CCTTTAGCATTGTTCCAAGTC-3‘ | reverse |
| GT198-8.1R*P | 5‘-GGCAGGTTTGACTATCCAGA-3‘ | reverse |
| GT198_3:UTR_SpeI_r* | 5‘-CCGGACTAGTATTCAAGCCTCAGATTTCTGC-3‘ | reverse |

* PCR primers used for genomic DNA derived from peripheral blood or buccal mucosa. Restriction

enzyme sites for *Xho*I, *Hind*III, *Xba*I or *Spe*I are added in primer.

^P^ Primers for formalin-paraffin-embedded tissue extracted genomic DNA
